# Review and evaluation of the methodological quality of the existing guidelines and recommendations for inherited neurometabolic disorders

**DOI:** 10.1186/s13023-015-0376-9

**Published:** 2015-12-30

**Authors:** Linda Cassis, Elisenda Cortès-Saladelafont, Marta Molero-Luis, Delia Yubero, Maria Julieta González, Aida Ormazabal Herrero, Carme Fons, Cristina Jou, Cristina Sierra, Esperanza Castejon Ponce, Federico Ramos, Judith Armstrong, M. Mar O’Callaghan, Mercedes Casado, Raquel Montero, Silvia Maria Meavilla Olivas, Rafael Artuch, Ivo Barić, Franco Bartoloni, Cinzia Maria Bellettato, Fedele Bonifazi, Adriana Ceci, Ljerka Cvitanović-Šojat, Christine I Dali, Francesca D’Avanzo, Ksenija Fumic, Viviana Giannuzzi, Christina Lampe, Maurizio Scarpa, Ángels Garcia- Cazorla

**Affiliations:** Neurology, gastroenterology pathology and clinical biochemistry Departments, IRP-HSJD and CIBERER, Barcelona, Spain; Department of Pediatrics, University Hospital Center Zagreb, Zagreb & University of Zagreb, School of Medicine, Zagreb, Croatia; Gianni Benzi Pharmacological Research Foundation, Valenzano, BA Italy; Department of Women and Children Health, Brains for Brain Foundation, Padova, Italy; Department of Clinical Genetics, Copenhagen University Hospital, Rigshospitalet, Copenhagen, Denmark; Department of Pediatric and Adolescent Medicine, Centre for Rare Diseases, Horst Schmidt Klinik Wiesbaden, Wiesbaden, Germany; Department of Women’s and Children’s Health, University of Padova, Padova, Italy

**Keywords:** Inherited neurometabolic disorders, Inborn errors of metabolism, Guidelines, Recommendations, AGREE II

## Abstract

**Background:**

Inherited neurometabolic disorders (iNMDs) represent a group of almost seven hundred rare diseases whose common manifestations are clinical neurologic or cognitive symptoms that can appear at any time, in the first months/years of age or even later in adulthood. Early diagnosis and timely treatments are often pivotal for the favorable course of the disease. Thus, the elaboration of new evidence-based recommendations for iNMD diagnosis and management is increasingly requested by health care professionals and patients, even though the methodological quality of existing guidelines is largely unclear. InNerMeD-I-Network is the first European network on iNMDs that was created with the aim of sharing and increasing validated information about diagnosis and management of neurometabolic disorders. One of the goals of the project was to determine the number and the methodological quality of existing guidelines and recommendations for iNMDs.

**Methods:**

We performed a systematic search on PubMed, the National Guideline Clearinghouse (NGC), the Guidelines International Network (G-I-N), the Scottish Intercollegiate Guideline Network (SIGN) and the National Institute for Health and Care Excellence (NICE) to identify all the published guidelines and recommendations for iNMDs from January 2000 to June 2015. The methodological quality of the selected documents was determined using the AGREE II instrument, an appraisal tool composed of 6 domains covering 23 key items.

**Results:**

A total of 55 records met the inclusion criteria, 11 % were about groups of disorders, whereas the majority encompassed only one disorder. Lysosomal disorders, and in particular Fabry, Gaucher disease and mucopolysaccharidoses where the most studied. The overall methodological quality of the recommendation was acceptable and increased over time, with 25 % of the identified guidelines strongly recommended by the appraisers, 64 % recommended, and 11 % not recommended. However, heterogeneity in the obtained scores for each domain was observed among documents covering different groups of disorders and some domains like 'stakeholder involvement' and 'applicability' were generally scarcely addressed.

**Conclusions:**

Greater efforts should be devoted to improve the methodological quality of guidelines and recommendations for iNMDs and AGREE II instrument seems advisable for new guideline development. The elaboration of new guidelines encompassing still uncovered disorders is badly needed.

**Electronic supplementary material:**

The online version of this article (doi:10.1186/s13023-015-0376-9) contains supplementary material, which is available to authorized users.

## Background

Inherited neurometabolic disorders (iNMDs) comprise almost seven hundred different rare diseases resulting from genetic defects, ranging from abnormal amino acid metabolism, impaired mitochondrial function, abnormal lipid trafficking to lysosomal storage diseases [1, 2]. The genetic defects affecting metabolic enzymes impact on the brain from birth and during the whole developmental period of childhood till adulthood, causing diverse neurological manifestations [3, 4].

INMDs, because of their rarity, still represent a challenge for many clinicians who are not able to properly diagnose, treat or follow-up affected patients. In addition, although effective treatments improving the life expectancy and/or quality of life exist for some iNMDs, they are often too expensive, not available in all countries or even administered too late.

Clinical practice guidelines (GLs) are commonly defined as “systematically developed statements to assist practitioner and patient decisions about appropriate health care for specific clinical circumstances” [5]. There are very few GLs and recommendations (RCs) that can assist patients, families, health professionals and support services to correctly manage iNMDs, and their methodological quality has never been systematically evaluated. For this reason, it may be also difficult for practitioners to choose the appropriate recommendations.

Because of the paucity of current information about most of these disorders, the European project "Inherited NeuroMetabolic Disease Information Network" (InNerMeD-I-Network, 2012 12 12, second Health Programme, http://www.innermed.eu) was launched with the aim of creating a network of information related to diagnosis and treatment of iNMDs. INMDs were classified in ten different categories, starting from previous existing classifications (http://www.orpha.net): (1) disorders of amino acids and organic acids; (2) purine, pyrimidine and neurotrasmitter metabolism diseases (3) disorders of carbohydrate metabolism; (4) disorders of lipid metabolism; (5) disorders of vitamin and non protein cofactor metabolism and transport; (6) disorders of porphyrin and hem metabolism; (7) disorders of mineral absorption and transport; (8) disorders of energy metabolism; (9) disorders of lysosomal and lysosomal-related organelles and (10) peroxisomal diseases. Recently, a new category of inborn errors of metabolism (IEMs) that currently includes more than one hundred diseases has been described: the defects of synthesis and remodeling of complex lipids [6].

In the present study we performed a systematic review of the literature to identify all published GLs and RCs about iNMDs. The aim of this study was to evaluate the number and the methodological quality of the GLs and RCs on iNMDs published from 2000 to 2015. To this purpose, we used the Appraisal of Guidelines, Research, and Evaluation II (AGREEII) instrument, a tool that evaluates the rigour and transparency in GL development and how well this process is reported [7–9]. A systematic analysis of the existing GLs and RCs for iNMDs may be useful for new guideline developers willing to follow a structured and rigorous elaboration methodology.

## Methods

### Search strategy

**Fig. 1:**
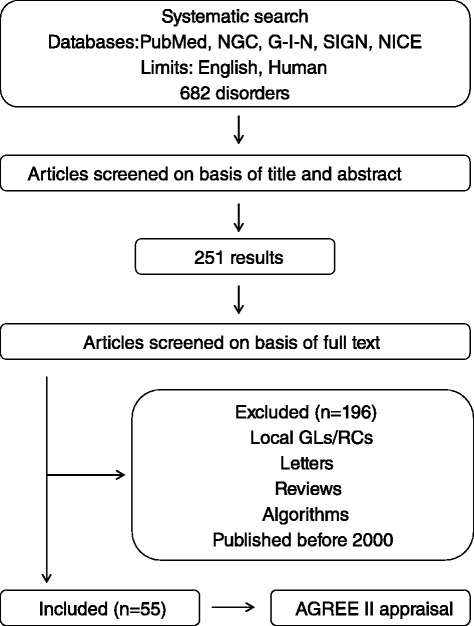
Flowchart of the strategy used to search and select GLs and RCs. A systematic search of the literature was performed in order to identify GLs and RCs encompassing 682 iNMDs. The documents were firstly selected on the basis of title and abstract (251 items). The analysis of the full text, as well as the application of the exclusion criteria, returned 55 unique documents that underwent AGREE II appraisal. GL: guideline; RC: recommendation; NGC: National Guideline Clearinghouse; G-I-N: Guideline International Network; SIGN: Scottish Intercollegiate Guideline Network; NICE: National Institute for Health and Care Excellence; AGREE: Appraisal of Guidelines, Research, and Evaluation

For each of the 682 identified iNMDs, grouped in ten categories, the following electronic databases related to GLs and RCs were systematically searched on February-March 2015: PubMed (http://www.ncbi.nlm.nih.gov/pubmed); the National Guideline Clearinghouse (NGC, http://www.guideline.gov); the Guidelines International Network (G-I-N, http://www.g-i-n.net); the Scottish Intercollegiate Guideline Network (SIGN, http://www.sign.ac.uk); the National Institute for Health and Care Excellence (NICE, http://www.nice.org.uk). Additional publications were included after manually checking the reference lists of the identified relevant documents. The strategy used to identify the GLs and RCs is shown in Fig. 1.

For PubMed database, the following searching strategy was used for each of the 682 diseases:

#1: “Disease" [Mesh]

#2: “Disease"

#3: #1 OR #2

#4: Recomm* OR Management OR update [TI/AB] OR Therapeutic* [TI/AB] OR treatment* OR guideline* OR consensus OR standard OR criterion [TI/AB])) OR ("Disease Management" [Mesh] OR "Therapeutics" [Mesh] OR "Health Planning Guidelines" [Mesh] OR "Guidelines as Topic" [Mesh] OR "Practice guidelines as Topic" [Mesh] OR "Review" [Publication Type] OR "Guideline" [Publication Type] OR "Practice Guideline" [Publication Type].

#5: #3 AND #4.

Search terms: disease, recommendation, management, update, therapeutic, treatment, guideline, consensus, standard, criterion, disease management, health planning guidelines, practice guideline, review.

### Inclusion and exclusion criteria

All the GLs and RCs published from 2000 to 2015 in the mentioned databases were selected by one of the authors (LC). Only English-written publications were considered. Duplicates of documents, which were found in more than one database, were manually removed from the selection. The initial search yielded unique records based on title and abstract (Fig. 1).

The final documents were then selected by two authors (LC and AGC) after reading the full text. First and updated versions of the same GLs and RCs were considered as distinct documents. Publications were excluded when the method used to formulate the GL or RC did not include a formal consensus process (such as the one reached through the Delphi Method or repetitive encounters) among independent professional groups or individuals. Moreover, GLs and RCs were not considered when they are applicable to one unique country. Reviews, algorithms and letters were also excluded. The selected unique records that responded to all the criteria for evaluation cover disorders related to:

**Table:** 

**A**	Amino acid and organic acid metabolism
**B**	Purine, pyrimidine and neurotrasmitter metabolism
**C**	Carbohydrate metabolism
**D**	Lipid metabolism
**E**	Vitamin and non protein cofactor metabolism and transport
**F**	Porphyrin and hem metabolism
**G**	Mineral absorption and transport
**H**	Energy metabolism
**I**	Lysosomal and lysosomal-related organelles
**J**	Peroxisomes

Defects of synthesis and remodeling of complex lipids were not considered in the search, because many of them are emerging disorders described in the last recent years.

For each study the following data were extracted: year of publication, name of the covered disease, number of authors, number of countries involved, country of origin of the authors, number of affiliations, and topics (screening, diagnosis, management and follow-up).

### Appraisal of guidelines and recommendations

The AGREE II instrument was used to assess the transparency, the methodological quality and the rigour of the selected GLs and RCs. It consists of 23 key items that address six quality domains: (1) scope and purpose, (2) stakeholder involvement, (3) rigor of development, (4) clarity of presentation, (5) applicability and (6) editorial independence. Two additional items concern the overall judgment of the GL (Overall Guideline Assessment) (Table 1).

**Table 1:** AGREE II instrument-domains and items

Domain	Item	
	Number	Content
1. Scope and purpose	1	The overall objective(s) of the guideline is (are) specifically described
	2	The clinical question(s) covered by the guideline is (are) specifically described
	3	The population to whom the guideline is mean to apply is specifically described
2. Stakeholder involvement	4	The guideline development group includes individuals from all relevant professional groups
	5	The views and preferences of the target population have been sought
	6	The target users of the guideline are clearly defined
3. Rigour of development	7	Systematic methods were used to search for evidence
	8	The criteria for selecting the evidence are clearly described
	9	The strengths and limitations of the body of evidence are clearly described
	10	The methods for formulating the recommendations are clearly described
	11	The health benefits, side effects, and risks have been considered in formulating the recommendations
	12	There is an explicit link between the recommendations and the supporting evidence
	13	The guideline has been externally reviewed by experts prior to its publication
	14	A procedure for updating the guideline is provided
4. Clarity of presentation	15	The recommendations are specific and unambiguous
	16	The different options for management of the condition or health issue are clearly presented
	17	Key recommendations are easily identifiable
5. Applicability	18	The guideline describes facilitators and barriers to its application
	19	The guideline provides advice and/or tools on how the recommendations can be put into practice
	20	The potential resource implications of applying the recommendations have been considered
	21	The guideline presents monitoring and/or auditing criteria
6. Editorial independence	22	The views of the funding body have not influenced the content of the guideline
	23	Competing interests of guideline development group members have been recorded and addressed
Overall assessment	1	Overall quality of this guideline
	2	Would you recommend this guideline for use?

The detailed criteria for each item are available in the user manual for AGREE II tool (http://www.agreetrust.org/). Briefly, for each document, the twenty-three items were rated on a 7-point scale (1–strongly disagree to 7–strongly agree) by two trained independent reviewers, experts in the field of IEMs and belonging to clinical, biochemical or genetic professional categories. One reviewer (LC) trained all the reviewers and rated all the documents, in order to provide a minimum variability between appraisals. A score of 1 was given when little or no relevant information was presented and a score of 7 was given when the statement met all criteria. Disagreement between reviewers (defined as ≥3 points difference in the score assigned by the appraisers to the same item) was resolved through consensus. To achieve consensus, the two reviewers shared the rationale for their appraisal and helped by the instructions provided by AGREE II instrument, they reached an agreement. According to AGREE II instructions, domain scores were calculated as (obtained score –minimum possible score)/(maximum possible score – minimum possible score) where "obtained score" is determined by summing up all the scores given by the appraisers for the individual items in a domain. All the final domain scores were entered into an Excel spreadsheet. Since the AGREE II manual does not provide guidance regarding how to interpret scores, to determine the grade of recommendation of the GL/RC we used a method previously applied by other authors with some minor modifications [10].

A GL or RC is “strongly recommended (SR)” when all the six item scores were ≥50 % or five item scores were ≥50 % and one item score was between 40 and 50 %. A guideline is “recommended (R)” if the overall quality assessment (OQA) score was ≥50 %. A guideline is “not recommended (NR)” if the OQA score was < 50 %.

The term GL and RC was assigned to the documents on the basis of the definition that the developers used to define the guidance they elaborated. However, all the articles underwent the same AGREE II appraisal process, regardless if they were GLs or RCs.

### Statistics

Data analysis was performed using the SPSS 22.0 software. The Kolmogorov-Smirnov test was applied to assess the data distribution. The ANOVA test was applied to compare score means of domains and individual questions between the established guideline groups. To study differences between GL groups, either parametric tests (if they follow a Gaussian distribution, Bonferroni Test) or non-parametric test (Games-Hewell) were applied. Pearson correlation test was applied to search for correlation between year of publication and number of GLs or RCs, and overall quality. Finally, the correlation between the quality of a GL and the number of authors, countries and affiliations involved was assessed. Statistical significance was defined as p < 0.05.

## Results

A total of 251 unique documents about detection or management of iNMDs are available so far and 87.3 % were published from 2000. The NGC defines GLs as *"statements that include recommendations intended to optimize patient care that are informed by a systematic review of evidence and an assessment of the benefits and harms of alternative care options*". Following this definition of GL and applying the inclusion and exclusion criteria shown in Fig. 1, only 21.9 % of the documents analyzed (*n* = 55) corresponded to GLs and RCs eligible for AGREE II appraisal*.*

### Characteristics of the GLs and RCs

Table 2 summarizes the information relative to the fifty-five identified GLs and RCs encompassing iNMDs.

**Table 2:** Characteristics of the guidelines and recommendations

Authors/titles	Year	Disorder	Authors (n)	Countries (n)	Affiliations (n)	Topics
Disorders of amino acid and other organic acid metabolism						
Arnold GL [22]	2008	3-methylcrotonyl CoA carboxylase deficiency	15	2	15	Diagnosis, management
Baumgartner MR [37]	2014	Methylmalonic and propionic acidemia	25	12	21	Screening, diagnosis, management, follow-up
Frazier D [38]	2014	Maple syrup urine disease	9	1	9	Management
Haberle J [19]	2012	Urea cycle disorders	15	4	14	Screening, diagnosis, management, follow-up
Kölker S [20]	2011	Glutaric aciduria type I	19	8	15	Screening, diagnosis, management
Kölker S [39]	2007	Glutaric aciduria type I	19	10	15	Screening, diagnosis, management
NIH CDP [40]	2001	Phenylketonuria	14	1	14	Screening, diagnosis, management, follow-up
Vockley J [41]	2014	Phenylketonuria	10	1	10	Screening, diagnosis, management, follow-up
Disorders of carbohydrate metabolism						
Barba-Romero MA [42]	2012	Pompe disease	13	1	13	Diagnosis, management, follow-up
Cochat P [43]	2012	Primary hyperoxaluria Type 1	18	6	16	Screening, diagnosis, management
Cupler EJ [44]	2012	Pompe disease	7	1	7	Diagnosis, management
Kishnani PS [45]	2014	Glycogen storage disease type I	15	1	8	Diagnosis, management
Kishnani PS [46]	2010	Glycogen Storage Disease Type III	16	1	10	Screening, diagnosis, management
Kishnani PS [47]	2006	Pompe disease	22	3	15	Screening, diagnosis, management, follow-up
Rake JP [48]	2002	Glycogen storage disease type I	6	4	4	Diagnosis, management, follow-up
Visser G [49]	2002	Glycogen Stoage Disease type I	8	5	6	Management
Winchester B [50]	2008	Pompe disease	29	17	25	Diagnosis
Disorders of vitamin and non protein cofactor metabolism and transport						
BCMSC [51]	2011	Cobalamin deciciency	unclear	1	unclear	Diagnosis, management, follow-up
Devalia V [52]	2014	Cobalamin and folate disorders	3	2	3	Screening, diagnosis, management
Disorders of porphyrin and haem metabolism						
Stein P [53]	2013	Porphyria	5	1	5	Diagnosis, management
Disorders of mineral absorption and transport						
Bacon BR [54]	2011	Hemochromatosis	5	2	5	Screening, diagnosis, management
BCMA [55]	2013	Hemochromatosis	Unclear	1	Unclear	Screening, diagnosis, management
EASL [56]	2012	Wilson Disease	8	Unclear	Unclear	Screening, diagnosis, management
Qaseem A [57]	2005	Hemochromatosis	6	1	5	Screening
Roberts EA [58]	2003	Wilson Disease	2	2	2	Diagnosis, management
Disorders of energy metabolism						
Angelini [23]	2006	Fatty acid mitochondrial disorders	6	4	5	Diagnosis, management
Arnold GL [59]	2009	Very long chain acyl-CoA dehydrogenase deficiency	14	2	14	Diagnosis, management
Finsterer J [60]	2009	Mitochondrial disorders	18	12	18	Diagnosis
Disorders of lysosomal and lysosomal-related organelles						
Andersson [61]	2005	Gaucher disease	10	1	10	Management, follow-up
Bennett RL [62]	2002	Fabry disease	9	1	8	Diagnosis, management, follow-up
Biegstraaten M [63]	2015	Fabry disease	34	15	29	Management
Charrow J [64]	2004	Gaucher disease	11	1	10	Diagnosis, management, follow-up
de Ru MH [65]	2011	Mucopolysaccharidosis type I	16	6	14	Management
Desnick RJ [66]	2003	Fabry disease	9	2	9	Diagnosis, management, follow-up
Eng CM [67]	2006	Fabry disease	13	4	11	Diagnosis, management, follow-up
Fahnehjelm KT [68]	2012	Mucopolysaccharidosis	7	5	5	Diagnosis, management
Giugliani R [69]	2007	Mucopolysaccharidosis VI	3	3	3	Diagnosis, management, follow-up
Grabowski GA [70]	2004	Gaucher disease	11	5	10	Diagnosis, management
Kaplan P [24]	2013	Gaucher disease	11	9	11	Diagnosis, management, follow-up
Kes VB [71]	2013	Fabry disease	16	1	11	Screening, diagnosis, management, follow-up
Laney DA [72]	2013	Fabry disease	9	1	9	Screening, diagnosis, management, follow-up
Langereis EJ [36]	2013	Mucopolysaccharidosis type I	17	8	15	Diagnosis, management, follow-up
Muenzer J [35]	2012	Mucopolysaccharidosis type II	11	6	11	Management, follow-up
Muenzer J [73]	2009	Mucopolysaccharidosis type I	12	6	Unclear	Diagnosis, management, follow-up
Ortiz A [74]	2008	Fabry disease	6	5	6	Diagnosis, management, follow-up
Patterson MC [25]	2012	Niemann-Pick disease type C	6	5	6	Diagnosis, management, follow-up
Peters C [75]	2003	Hematopoietic cell transplantation for IMDs	Unclear	Unclear	2	Management, follow-up
Scarpa M [34]	2011	Mucopolysaccharidosis type II	26	14	25	Screening, diagnosis, management
Solanki GA [76]	2012	Mucopolysaccharidosis type VI	13	4	13	Diagnosis, management, follow-up
Terryn W [77]	2013	Fabry disease	9	5	9	Screening, diagnosis, management, follow-up
Vellodi A [78]	2001	Gaucher disease	8	6	8	Maganement, follow-up
Vom Dahl S [79]	2006	Gaucher disease	7	4	7	Follow-up
Wang RY [80]	2011	Lysosomal storage diseases	4	1	5	Screening, diagnosis, management, follow-up
Weinreb NJ [26]	2004	Gaucher disease	25	14	24	Diagnosis, follow-up
Wraith JE [81]	2009	Niemann-Pick disease type C	13	10	13	Screening, diagnosis, management, follow-up

*NIH CDP* National institutes of health consensus development panel, *BCMSC* British Columbia medical services Commission, *BCMA* British Columbia medical association, *EASL* European association for study of liver

**Fig. 2:**
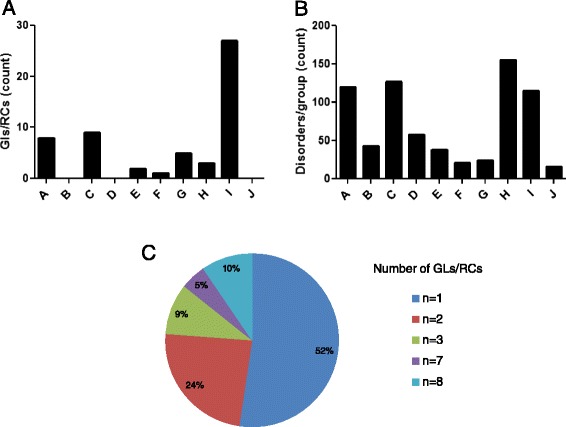
Characteristics of the identified GLs and RCs. **a** Number of the GLs and RCs selected for each group of disorders. **b** Number of disorders belonging to each group. **c** Percentage of disorders for which 1 to 8 different GLs and RCs were identified. A: amino acid and organic acid metabolism (*n* = 8); B: Purine, pyrimidine and neurotrasmitter metabolism (*n* = 0); C: carbohydrate metabolism (*n* = 9); D: lipid metabolism (*n* = 0); E: vitamin and non protein cofactor metabolism and transport (*n* = 2); F: porphyrin and hem metabolism (*n* = 1); G: mineral absorption and transport (*n* = 5); H: energy metabolism (*n* = 3); I: lysosomal and lysosomal-related organelles (*n* = 27); J: peroxisomes (*n* = 0)

GLs and RCs were identified for almost all the subtypes of iNMDs, except for disorders of purine, pyrimidine and other neurotransmitter metabolism (group B), lipid metabolism (group D) and peroxisomal disorders (group J). The groups of disorders highly differed in the number of associated GLs and RCs, since documents encompassing disorders of vitamin and non protein cofactor metabolism and transport (group E), porphirin and hem metabolism (group F) and energy metabolism (group H) were covered by a small number of GLs and RCs (*n* = 2, *n* = 1, *n* = 3, respectively). In contrast, the disorders associated with lysosomal and lysosomal-related organelles were the most encompassed by GLs and RCs (*n* = 27, Fig. 2a). These numbers did not necessarily correlate with the number of disorders in each group. For instance, the number of disorders associated to lysosome and lysosomal-related organelles (group I, *n* = 115) was lower than the number of disorders of energy metabolism (group H, *n* = 155), even though the number of GLs and RCs was ninefold higher in the former group (*n* = 27 and *n* = 3, respectively, Fig. 2b).

Six of the identified documents were about groups of disorders (11 %), while the remaining 49 pubblications focused on one unique disorder and globally covered twenty different diseases (Table 2). Moreover, 52 % of the considered pathological conditions were covered by only one document. In contrast, GLs and RCs about Gaucher disease, Fabry disease, and mucopolysaccharidoses (MPSs) were reported in seven (Gaucher disease) and eight (Fabry disease and MPSs) different publications (Additional file 1: Table S1 and Fig. 2c).

**Fig. 3:**
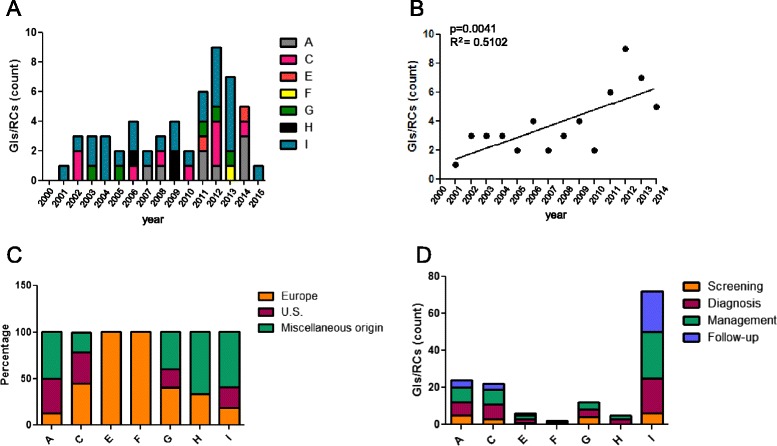
Publication date, origin and topics of the identified GLs and RCs. **a** Number of GLs and RCs published between 2000 and 2015. **b** Correlation between year of publication from 2000 to 2014 and number of GLs or RCs, linear regression. **c** Country of origin of the GLs and RCs. For each document, the countries of origin of all the authors were analyzed and GLs and RCs were classified as: from Europe, from USA or from miscellaneous origin (different continents or different American countries). Data are expressed as relative percentage referred to total GLs and RCs covering the same group of disorders. **d** Topics covered by the identified GLs and RCs. A: amino acid and organic acid metabolism (*n* = 8); C: carbohydrate metabolism (*n* = 9); E: vitamin and non protein cofactor metabolism and transport (*n* = 2); F: porphyrin and hem metabolism (*n* = 1); G: mineral absorption and transport (*n* = 5); H: energy metabolism (*n* = 3); I: lysosomal and lysosomal-related organelles (*n* = 27)

Although the number of new documents/year has been quite stable all over the last first decade, the overall frequency of GLs and RCs about iNMDs has significantly increased over time (Fig. 3a and b).

The analysis of the country of origin of the authors revealed that 29.1 % of the GLs and RCs were from Europe, 23.6 % from USA and the remaining 42.3 % documents were the result of a collaboration among authors coming from different continents or American countries (miscellanueous origin) (Fig. 3c).

Finally, 87.3 % of the GLs and RCs covered more than one topic and dealt principally with the diagnosis (80 %) and management (91 %) of the disease. Screening and follow-up-related issues were encompassed by 34 % and 55 % of the documents, respectively. No GLs and RCs have been developed for the follow-up of disorders of porphirin and hem metabolism (group F), mineral absorption and transport (group G) and energy metabolism (group H, Table 2 and Fig. 3d).

**Fig. 4:**
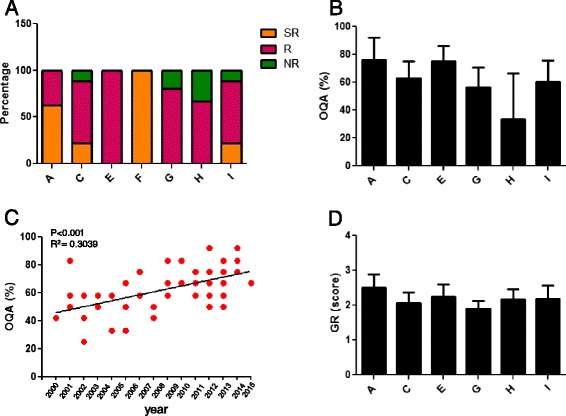
Overall quality of the identified GLs and RCs. **a** Overall recommendation: percentage of strongly recommended (SR), recommended (R) and not recommended (NR) GLs and RCs. Data are expressed as relative percentage referred to total GLs and RCs covering the same group of diseases. **b** Overall quality assessment (OQA) of GLs and RCs. Data are presented as mean ± SD. **c** Correlation between OQA and year of publication, linear regression. **d** Grade of recommendation (GR) of GLs and RCs (scores from 1 = not recommended to 3 = recommended without modifications). Data are presented as mean ± SD. A: amino acid and organic acid metabolism (*n* = 8); C: carbohydrate metabolism (*n* = 9); D: lipid metabolism; E: vitamin and non protein cofactor metabolism and transport; G: mineral absorption and transport (*n* = 5); H: energy metabolism (*n* = 3); I: lysosomal and lysosomal-related organelles (*n* = 27)

The level of recommendation of the GLs and RCs was determined by the number of items scoring ≥50 %. The AGREE II analysis revealed that 25 % of the identified GLs were strongly recommended (SR), 64 % recommended (R), and 11 % not recommended (NR, Fig. 4a). All the documents had at least one item scoring ≥50 % (Table 3). We excluded from the subsequent statistical analysis the unique document relative to disorders of porphirin and hem metabolism (group F).

**Table 3:** Results of AGREE II appraisal for all the identified guidelines and recommendations

Authors	OR	D1	D2	D3	D4	D5	D6	OQA	GR
Disorders of amino acid and other organic acid metabolism									
Arnold GL	R	67	44	52	61	29	0	50	2
Baumgartner MR	SR	94	86	95	94	46	50	92	3
Frazier D	SR	78	92	83	97	65	58	92	2.5
Haberle J	SR	83	89	90	89	58	96	92	3
Kölker S-2011	SR	56	58	83	92	63	100	75	2.5
Kölker S-2007	R	67	36	91	89	29	100	75	2.5
NIH CDP	SR	89	86	86	81	73	4	58	2.5
Vockley J	R	89	31	60	78	48	54	75	2
Disorders of carbohydrate metabolism									
Barba-Romero MA	R	80	61	53	83	31	50	75	2
Cochat P	R	72	28	27	92	31	33	58	2.5
Cupler EJ	R	64	64	46	67	41	63	75	2
Kishnani PS-2014	SR	86	61	47	100	58	63	75	2.5
Kishnani PS-2010	R	89	61	50	83	48	33	67	2
Kishnani PS-2006	SR	83	56	44	56	50	75	67	2
Rake JP	R	94	17	27	92	25	0	58	2
Visser G	R	83	19	23	83	23	0	50	2
Winchester B	NR	72	36	14	53	25	67	42	1.5
Disorders of vitamin and non protein cofactor metabolism and transport									
BCMSC	R	94	31	51	61	27	0	67	2.5
Devalia V	R	83	33	71	94	67	83	83	2
Disorders of porphyrin and hem metabolism									
Stein P	SR	89	81	56	89	56	50	75	2.5
Disorders of mineral absorption and transport									
Bacon BR	R	56	44	75	83	46	17	67	2
BCMA	R	94	8	13	81	27	0	58	2
EASL	R	67	28	49	86	35	38	67	2
Qaseem A	NR	100	36	27	50	23	42	33	1.5
Roberts EA	R	67	17	32	78	33	0	58	2
Disorders of energy metabolism									
Angelini	NR	64	8	36	31	8	21	33	1.5
Arnold GL	R	86	67	61	72	38	0	67	2
Finsterer J	R	75	53	48	72	19	0	58	2.5
Disorders of lysosomal and lysosomal-related organelles									
Andersson	R	83	33	29	67	25	0	58	2.5
Bennett RL	SR	92	86	69	78	52	46	83	3
Biegstraaten M	SR	64	81	65	64	42	71	67	2
Charrow J	R	81	56	43	69	33	13	58	2
de Ru MH	SR	89	50	63	89	54	58	75	2.5
Desnick RJ	NR	64	22	40	53	48	46	42	2
Eng CM	R	92	42	45	89	46	0	67	2.5
Fahnehjelm KT	R	75	58	32	81	38	50	75	2.5
Giugliani R	R	75	56	56	67	29	38	58	2
Grabowski GA	R	72	33	27	56	33	0	50	2
Kaplan P	R	78	39	29	78	38	100	83	2
Kes VB	R	61	33	29	56	21	0	50	2
Laney DA	R	94	92	46	78	46	83	67	2.5
Langereis EJ	R	86	72	58	53	23	46	58	2
Muenzer J-2012	R	92	53	33	69	40	92	50	2.5
Muenzer J-2009	R	69	36	28	69	42	50	58	2
Ortiz A	R	58	31	40	61	23	92	50	2.5
Patterson MC	R	78	28	38	78	42	4	67	2
Peters C	NR	64	39	25	33	13	0	25	1.5
Scarpa M	SR	61	75	61	89	42	58	75	2.5
Solanki GA	R	72	39	24	61	54	100	50	1.5
Terryn W	SR	81	44	67	78	58	54	67	2.5
Vellodi A	NR	83	61	30	72	17	0	42	1.5
Vom Dahl S	R	89	36	54	56	31	83	50	2
Wang RY	SR	92	64	53	92	67	92	83	2.5
Weinreb NJ	R	67	39	30	67	17	4	50	2
Wraith JE	R	83	28	46	83	42	0	83	3

*OR* Overall recommendation, *D* Domain, *OQA* Overall quality assessment, *GR* Grade of recommendation, *NIH CDP* National institutes of health consensus development panel, *BCMSC* British Columbia medical services commission, *BCMA* British Columbia medical association, *EASL* European association for study of liver, *SR* Strongly recommended, *R* Recommended, *NR* Not recommended domain scores were calculated as described in Methods

### Overall quality assessment of GLs and RCs

In this domain, the appraiser is invited to judge the overall quality of the GL and to indicate whether she/he would recommend it for use.

The range and mean ± SD of the overall quality assessment (OQA) score for this domain were 25–92 % and 63 % ± 15 % (Table 3 and Fig. 4b). Interestingly, although we did not find any statistically significant differences among groups, the OQA of the GLs and RCs about iNMDs increased over the years (Fig. 4c).

A further 3-point scale (1 = not recommended, NR; 2 = recommended with modifications, R + M, and 3 = recommended, R) was introduced in the analysis, providing an additional overall judgment on whether the GLs or RCs should be recommended for use (Table 3). For this item, the range and mean ± SD of the overall score were 1.5–3 % and 2.2 % ± 0.4 %. Almost all the GLs and RCs were recommended, since they scored ≥2, except for six documents (11 %) that were not recommended. On the other hand, appraisers considered that 83 % of the GLs and RCs required modifications. Of note, two out of three documents recommended without modifications by both appraisers belonged to the group of amino acid and organic acid metabolism (group A).

**Fig. 5:**
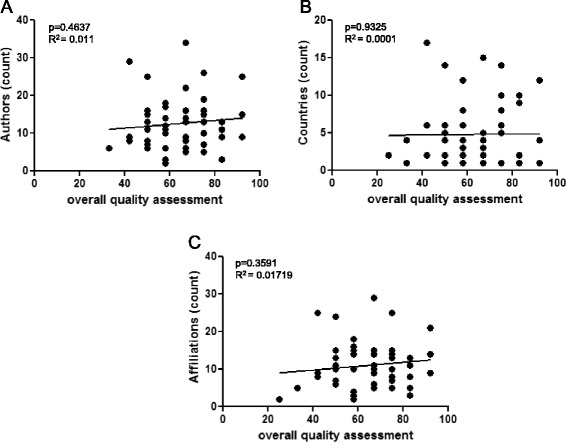
Correlation between quality of the GLs/RCs and number of authors, countries or affiliations. Linear regression analysis was used to determine whether the overall quality assessment (OQA) of a GL could depend on the number of authors (**a**), countries (**b**) or affiliations (**c**) involved in its elaboration

The quality of a GL is supposed to improve when it is developed by experienced experts coming from different countries, or distinct professional categories. For this reason, we next wondered whether the overall quality of the GLs and RCs could depend on the number of authors, countries or affiliations to which authors belong. Figure 5a-c shows that a direct correlation between the OQA and these parameters could not be determined, although a non statistically significant increase in the OQA was observed for higher number of authors and affiliations.

### Domain 1: scope and purpose

**Fig. 6:**
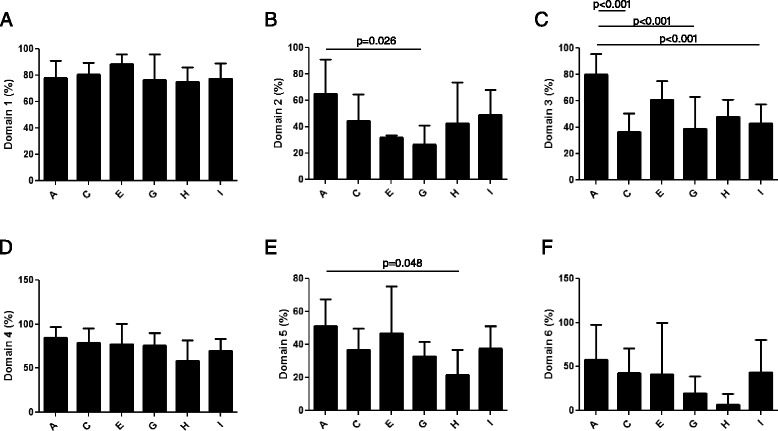
Results of AGREE II appraisal for domains 1–6. The identified GLs and RCs were scored on a 7-point scale for the 23 items belonging to domain 1 (**a**), domain 2 (**b**), domain 3 (**c**), domain 4 (**d**), domain 5 (**e**) and domain 6 (**f**). Data are presented as mean ± SD of values obtained by each group of disorders in each domain. A: amino acid and organic acid metabolism (*n* = 8); C: carbohydrate metabolism (*n* = 9); E: vitamin and non protein cofactor metabolism and transport (*n* = 2); G: mineral absorption and transport (*n* = 5); H: energy metabolism (*n* = 3); I: lysosomal and lysosomal-related organelles (*n* = 27)

This domain considers whether the overall objectives of the GL, the health questions covered by the GL and the population whom the GL is directed to are specifically described. The range and mean ± SD of the overall score for this domain were 56–100 % and 78 % ± 18 %. Table 3 and Fig. 6a report the score recorded for each GL and the overall score obtained by the different groups of disorders. The scores were comparable for all the considered groups. None of the GLs or RCs scored <50 %.

### Domain 2: stakeholder involvement

**Fig. 7:**
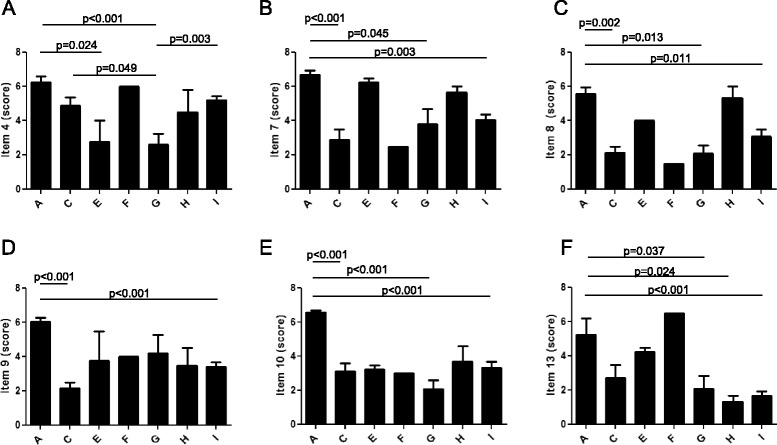
Results of AGREE II appraisal for individual items. Scores obtained by the identified GLs and RCs on a 7-point scale for item 4 (**a**), item 7 (**b**), item 8 (**c**), item 9 (**d**), item 10 (**e**) and item 13 (**f**). Data are presented as mean ± SD of values obtained by each group of disorders in each item. A: amino acid and organic acid metabolism (*n* = 8); C: carbohydrate metabolism (*n* = 9); E: vitamin and non protein cofactor metabolism and transport (*n* = 2); G: mineral absorption and transport (*n* = 5); H: energy metabolism (*n* = 3); I: lysosomal and lysosomal-related organelles (*n* = 27)

This domain evaluates whether the GL development group includes individuals from all relevant professional groups, the views and preferences of the target population have been sought, and the target users of the GL are clearly defined. The range and mean ± SD of the overall score for this domain were 8–92 % and 48 % ± 23 % (Table 3 and Fig. 6b). Almost half of the overall GLs and RCs, as well as all the documents related to disorders of vitamin and non protein cofactor metabolism and transport (group E) and mineral absorption and transport (group G), scored < 50 %. In addition, we also found statistically significant differences in the scores obtained by the different groups in item 4, which refers to the composition of the GL development group (Fig. 7a). For item 5, referring to the involvement of the target population in the elaboration of the GLs, 75 % of the documents had a score between 1 and 2, indicating that most of them did not consider patients or public in the process (data not shown).

### Domain 3: rigour of development

This domain relates to the method used to search and select the evidence and to formulate the GL. It also focuses on the health benefits, side effects, and risks that should be considered when formulating the recommendations. The range and mean ± SD of the overall score for this domain were 13–95 % and 48 % ± 22 %. (Table 3 and Fig. 6c).

Whereas all the documents for disorders of amino acid and organic acid metabolism (group A) obtained a mean score ≥50 %, thirty-one GLs and RCs (56 %) presented scores < 50 % for this domain.

The statistical analysis of the distinct items revealed that the seven groups of disorders obtained different scores in items 7, 8, 9, 10 and 13 (Fig. 7b-f). Item 13 considers whether the GL has been externally reviewed by experts prior to its publication. For this items, only 30 % of the documents obtained a score > 3, indicating that the majority did not undergo an external revision prior to submission for publication or did not provide sufficient information. Item 14 judges if a procedure for GL updating has been established and 73 % of the documents provided scarce information (score ≤3, data not shown).

### Domain 4: clarity of presentation

This domain examines whether the RCs are specific and unambiguous, the different options for management are clearly presented and key RCs are easily identifiable. The range and mean ± SD of the overall score for this domain were 31–100 % and 74 % ± 20 %. Table 3 and Fig. 6d show that all the groups of disorders presented a comparable score for this domain and no statistically significant difference was observed. Only two documents (3.6 %), belonging to the disorders of energy metabolism (group H) and lysosomal and lysosomal-related organelles (group I), presented scores < 50 %.

### Domain 5: applicability

This domain examines whether the GL describes facilitators and barriers to its application, explains how the RCs could be put into practice and considers the potential resource implications of applying the RCs. The range and mean ± SD of the overall score for this domain were 8–73 % and 39 % ± 16 % (Table 3 and Fig. 6e). Collectively, 76 % of all the identified documents had a score < 50 % and all the GLs and RCs about mineral absorption and transport (group G) and energy metabolism (group H) obtained scores < 50 %. Analyzing the distinct items, we observed that 38, 58, 80 and 24 % of the documents achieved scores ≤3 for item 18, 19, 20, and 21, respectively. In particular, for item 20 (potential cost impact of the GL) the majority of the identified GLs did not sufficiently consider the cost effectiveness or implications for budget of applying the recommendations (data not shown).

### Domain 6: editorial independence

This domain assesses whether the views of the funding body have not influenced the content of the GL and whether the competing interests of GL development group members have been recorded and addressed. The range and mean ± SD of the overall score for this domain were 0–100 % and 41 % ± 35 %. (Table 3 and Fig. 6f). No statistically significant difference was observed among groups. Only 47 % of total GLs and RCs had a score ≥50 % and none of the documents related to disorders of energy metabolism (group H) and mineral absorption and transport (group G) reached this score for this domain.

### Gaucher disease and Fabry disease were the most studied diseases

We next focused on two lysosomal storage disorders, Gaucher disease and Fabry disease, encompassed by seven and eight different GLs and RCs, respectively. Gaucher disease is an inherited disorder with an estimated birth prevalence of 1:40,000 to 1:60,000, caused by deficient activity of the lysosomal enzyme glucocerebrosidase [11, 12]. Fabry disease affects approximately 1:40,000-170,000 individuals and it is caused by a deficiency of the lysosomal hydrolase α- galactosidase A [13].

**Fig. 8:**
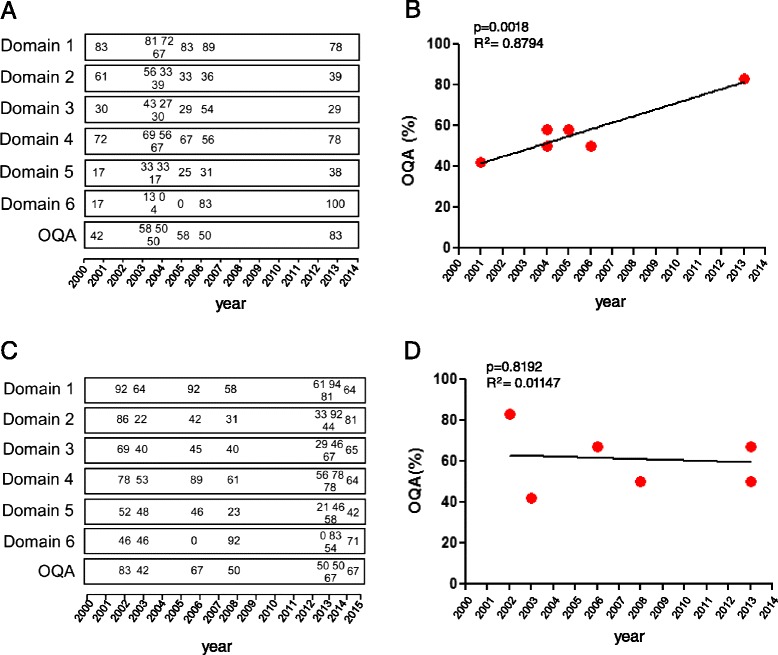
Results of AGREE II appraisal for Gaucher disease and Fabry disease. GLs and RCs for Gaucher disease (**a**) and Fabry disease (**c**) were scored on a 7-point scale for the 23 items belonging to domain 1–6. Numbers indicate the scores obtained in each domain by GLs and RCs grouped by year of publication. Correlation between overall quality assessment (OQA) and year of publication was determined for all existing GLs and RCs for Gaucher disease (**b**) and Fabry disease (**d**), linear regression

The aim was to determine whether the quality and rigour of the GLs and RCs improved over time. For this purpose, the scores referring to domains 1–6 and to the OQA domain were analyzed. For Gaucher disease, we did not find an increase in the scores referring to domains 1–6. However, a statistically significant increase in the OQA was observed (Additional file 1: Table S1 and Fig. 8a and b).

By contrast, for Fabry disease no improvement was observed in none of the appraised domains (Fig. 8c and d).

## Discussion

This is the first systematic estimation of the number and methodological quality of all existing GLs and RCs about iNMDs, which have been reclassified inside the activities of the EU funded project InNerMeD-I- Network. The project InNerMeD-I-Network is aimed to group the most vast multidisciplinary network in order to collect, exchange and share validated information among scientific communities, health professionals, patients, patient associations, public health authorities, pharmaceutical companies and other interested parties. One of the focus of this project is the analysis of existing and the elaboration of new GLs and RCs that will provide practical support for the diagnosis and treatment of iNMDs.

To this aim, for the analysis of existing GLs and RCs for iNMDs, we have selected the AGREE II instrument, considered one of the best choice to conduct a comprehensive GL appraisal [7, 14]. Importantly, AGREE II tool does not evaluate the medical/scientific content validity or the evidence base underlying a RC, but the rigour and transparency applied during GL development [15]. Thus, the performed analysis can be considered as a valuable tool for the elaboration of new GLs and RCs for iNMDs, since it provides methodological models for GL developers. On the other hand, it also offers a precise and updated picture about the existing GLs for iNMDs. This review could be useful for practical clinical purposes but also as an overview to detect what disorders do not currently have any official guide.

AGREE II scores indicate that the overall methodological quality of the GLs and RCs for selected iNMDs is acceptable (although very few of them obtained a score ≥50 % in at least five items), and increasing over time. In addition, in all the appraised GLs and RCs the authors sufficiently describe the overall objectives, the covered health questions and the population whom they are directed.

Similarly, the appraisers judged that for most of the documents, the RCs were sufficiently specific, unambiguous, and easily identified, and that the different options for diagnosis or management were clearly presented. The availability of identifiable information facilitates the task of the health professionals when choosing the proper guidance.

We choose the year 2000 as lower limit for GL and RC search because we consider that from this year onwards experts in iNMDs have acquired more awareness about the necessity to provide guidance, as well as to collaborate and create networks. This may be due to the fact that in the last 15 years the number of patients who have been successfully diagnosed for iNMDs increased, also thanks to the availability of more precise and reliable diagnostic techniques. In this regard, we observed a substantial increase in the number of GLs and RCs for iNMDs, especially in the last few years.

Interestingly, only 23 % of the 251 documents identified in the first search met the inclusion criteria and we often observed a widespread misuse of the term "guideline" that was utilized for systematic reviews, algorithms or letters. Indeed, until now the most common way of providing an overview or global knowledge about a particular disease or group of disorders in the field of iNMDs was by elaborating extensive systematic reviews of the published literature. The resultant documents were often used by health professionals as GLs or RCs. In the last years, the tendency changed and groups of experts meet with the exclusive purpose of writing well-structured evidence-based GLs [16, 17].

However, we could not find GLs and RCs for most of iNMDs, although disorders including Pompe disease, Gaucher disease, Fabry disease and MPSs were covered by at least three GLs or RCs. This disproportionate distribution of GLs and RCs could imply that guidance is prevalently provided for potentially treatable disorders, for which fully tested or still experimental therapies already exist. In this respect, we also observed that 50-100 % of the GLs and RCs for Pompe disease, Gaucher disease, Fabry disease and MPSs were partially or totally funded by pharmaceutical companies, which may be particularly interested in the dissemination of RCs that promote the use of their own treatments. This observation may also entails that GL development is a costly process that may require external funding to be realized and may explain why most of the iNMDs still do not have guidance for their diagnosis or treatment.

On the other hand, it has to point out that often GLs and RCs not even exist for several treatable disorders, including different vitamin (thiamine, riboflavine, biotine, vitamin E)-responsive diseases, the glucose transporter type 1 (Glut1) deficiency, or the cerebrotendinous xanthomatosis (CXT), among others. In this case, lack of GLs could be ascribed to a still insufficient information about the phenotype and the progression of the disorder, or the correct treatment requirement.

However, two different European networks are currently working on the elaboration of GLs and RCs for treatable homocystinurias and neurotrasmitter defects (unpublished data).

The rarity of iNMDs may also explain the lack of available GLs for their screening, diagnosis, management or follow-up. In fact, few clinical trials exist and the published evidence often consists of isolated case reports and is not sufficient to elaborate a GL. Similarly, expert opinion is often inadequate in the case of ultra-rare disorders, because clinicians can see an individual condition only rarely in a career [18]. Nevertheless, the knowledge in the field of iNMDs has enormously increased in the last decades. Thus, the establishment of international networks composed by experts encompassing all the health specialties related to iNMD disorders could dramatically enhance the number of GLs for practice. In particular, more attention should be paid to disorders for which therapeutic possibilities already exist, in order to establish the bases for a proper treatment or follow-up of the patients.

Some recent GLs, such as those about urea cycle disorders (UCDs) or organic acidurias (OA) among others, have been created in the context of European-International projects funded by the European Commission, where consortiums composed by many countries and expert centers were formed in order to elaborate specific plans for GL development [19, 20]. This strategy could explain why GLs and RCs covering disorders of amino acid and organic acid metabolism obtained the highest scores in almost all the domains. Indeed, our results show that although the correlation was not statistically significant, the overall quality of the GLs and RCs was higher when the number of authors or affiliations involved in its development increased.

The AGREE II analysis revealed that the elaboration of most of the selected GLs did not involve the target population (patients or public). This could be due to different factors, such as the difficulty to identify and recruit patients or representatives of the public or the discrepancy between the perspectives of expert and non-expert members. In addition, the target population often does not have familiarity with the scientific and medical terminology [21]. Nonetheless, an effort should be done to increase the collaboration between experts and patients or public, by providing them with scientific support, training and mentoring. This could lead to more relevant and understandable GLs or to the production of additional material, such as quick reference guides or leaflets that could assist patients and families along the course of the disease.

Our data also showed that the majority of the GLs and RCs were published without an external review prior to submission for publication. The GL revision by one or more external experts working in the same subject area would likely improve the methodological quality of the recommendations. In fact, external reviewers coming from different countries or medical fields could provide a more comprehensive view about diagnosis or management options for iNMDs.

On the other hand, an expert external advice prior to the elaboration of the guidance would be certainly valuable, in order to ensure the adoption of the most rigorous and structured possible methodology.

We observed that 73 % of the GLs and RCs did not provide a procedure for their updating, and some disorders were covered by only one document published several years ago [22, 23]. On the other hand, four of the documents are revised version of previously published GLs and RCs [20, 24–26]. The updated version of previous GLs and RCs may present an improve in the OQA [24], or in the overall recommendation grade [20]. However, the small number of updated documents we identified with our search is not sufficient to determine whether the methodological quality of a guidance increases in its later versions. The updating of a GL should collect all the new evidence reported as well as information about the development of new technologies in diagnosis and treatment, thus reducing the variability among the documents published about the same disorders. However, very little information is available so far to indicate when a GL should be updated [27–29]. In this respect, we observed that the time between the first and the updated versions of the same GLs and RCs for iNMDs ranged between 3 and 9 years.

The frequency of iNMDs varies among different populations, and higher rate of consanguinity normally results in a significantly higher incidence of the disease. For example, the frequency of methilmalonic acidemia (MMA) is higher in Saudi Arabia, whereas glutaric aciduria type 1 (GA1) is more recurrent in the Old Order Amish in United States. This heterogeneous distribution of iNMD might imply that different strategies should be adopted by each country according to its possibilities. Indeed, because of the local economic conditions, some treatments, medical instruments or support infrastructure may be available in one country, but eventually not in another one [30, 31]. In this regard, none of the appraised documents referred to suitable alternatives for more vulnerable regions or countries, although an assessment of the implementation and adaptation of GLs for UCDs has been recently reported [31].

In addition, most of the analyzed documents did not consider neither the economical impact of applying the GL, probably because it may be difficult to predict costs and benefits, especially for long-term treatments [16]. Finally, none of the appraised GLs and RCs evaluated the risks versus benefits related to different social, economic or geographic realities.

This systematic search may present some limitations. First, we cannot exclude that we may have overlooked important GLs and RCs. Moreover, we only included documents in English, so GLs and RCs in other languages were not considered. Second, GLs were reviewed by two different appraisers and although a consensus was reached in case of discrepancy, they might have different level of understanding of the AGREE II instrument. In addition, since a reference cannot be provided by the AGREE II instrument, the perception that the appraiser had of the quality of a GL may have varied for each document and could be influenced by the comparison with the previous one.

## Conclusions

Our analysis indicates that more documents are needed to encompass all the iNMDs that still lack guidance for their screening, diagnosis, treatment or follow-up. Considering AGREE II as a valuable tool for GL development, we observed that many of the existing GLs and RCs do not respond to the proposed criteria. In particular, new GL developers should take greater account of aspects that are still too overlooked. First of all, a GL should contain accurate information about how the evidence is searched, selected, validated and supporting the RC. Moreover, GL developers should consider the associated health benefits, side effects, and risks. In this regard, we also emphasize the necessity to externally review and update the GLs and to adapt them to the different social, economic and cultural realities. Finally, patients and society should be called to participate in the development of new RCs.

## Additional file

Additional file 1: Table S1. Results of AGREE II appraisal for guidelines and recommendations for the most frequently covered iNMDs. (DOC 70 kb)
