# Erratum to: Review and evaluation of the methodological quality of the existing guidelines and recommendations for inherited neurometabolic disorders

**DOI:** 10.1186/s13023-016-0431-1

**Published:** 2016-11-03

**Authors:** Linda Cassis, Elisenda Cortès-Saladelafont, Marta Molero-Luis, Delia Yubero, Maria Julieta González, Aida Ormazábal, Carme Fons, Cristina Jou, Cristina Sierra, Esperanza Castejon Ponce, Federico Ramos, Judith Armstrong, M. Mar O’Callaghan, Mercedes Casado, Raquel Montero, Silvia Meavilla-Olivas, Rafael Artuch, Ivo Barić, Franco Bartoloni, Cinzia Maria Bellettato, Fedele Bonifazi, Adriana Ceci, Ljerka Cvitanović-Šojat, Christine I. Dali, Francesca D’Avanzo, Ksenija Fumic, Viviana Giannuzzi, Christina Lampe, Maurizio Scarpa, Ángels Garcia-Cazorla

**Affiliations:** 1 Neurology, gastroenterology pathology and clinical biochemistry Departments, IRP-HSJD and CIBERER, Barcelona, Spain; 2 Department of Pediatrics, University Hospital Center Zagreb, Zagreb & University of Zagreb, School of Medicine, Zagreb, Croatia; 3 Gianni Benzi Pharmacological Research Foundation, Valenzano, BA Italy; 4 Department of Women and Children Health, Brains for Brain Foundation, Padova, Italy; 5 Department of Clinical Genetics, Copenhagen University Hospital, Rigshospitalet, Copenhagen, Denmark; 6 Department of Pediatric and Adolescent Medicine, Centre for Rare Diseases, Horst Schmidt Klinik Wiesbaden, Wiesbaden, Germany; 7 Department of Women’s and Children’s Health, University of Padova, Padova, Italy

## Erratum

Unfortunately, the original version of this article [1] contained an error. The author list displayed the following author names incorrectly. Aida Ormazábal was listed as Aida Ormazabal Herrero, Silvia Meavilla-Olivas as Silvia Maria Meavilla Olivas and Ángels Garcia-Cazorla as Ángels Garcia- Cazorla. This has been corrected in the original article and can also be seen correctly above.
