# Supplementary material for: Review and evaluation of the methodological quality of the existing guidelines and recommendations for inherited neurometabolic disorders
Source: Orphanet J Rare Dis. 2015 Dec 30;10:164. doi: 10.1186/s13023-015-0376-9 (PMC4696316; doi:10.1186/s13023-015-0376-9)
Supplement: Additional file 1: Table S1. — Results of AGREE II appraisal for guidelines and recommendations for the most frequently covered iNMDs. (DOC 70 kb) [file 13023_2015_376_MOESM1_ESM.doc]

**Additional file 1: Table S1. Results of AGREE II appraisal for guidelines and recommendations for the most frequently covered iNMDs**

|  | | | | | | | | | |
| --- | --- | --- | --- | --- | --- | --- | --- | --- | --- |
| **Authors** | **OR** | **D1** | **D2** | **D3** | **D4** | **D5** | **D6** | **OQA** | **GR** |
| **Pompe disease** | | | | | | | | | |
| [Barba-Romero MA](http://www.ncbi.nlm.nih.gov/pubmed/?term=Barba-Romero MA%5BAuthor%5D&cauthor=true&cauthor_uid=22492103) | R | 80 | 61 | 53 | 83 | 31 | 50 | 75 | 2 |
| [Cupler EJ](http://www.ncbi.nlm.nih.gov/pubmed/?term=Cupler EJ%5BAuthor%5D&cauthor=true&cauthor_uid=22173792) | R | 64 | 64 | 46 | 67 | 41 | 63 | 75 | 2 |
| [Kishnani PS](http://www.ncbi.nlm.nih.gov/pubmed/?term=Kishnani PS%5BAuthor%5D&cauthor=true&cauthor_uid=16702877) | SR | 83 | 56 | 44 | 56 | 50 | 75 | 67 | 2 |
| Winchester B | NR | 72 | 36 | 14 | 53 | 25 | 67 | 42 | 1.5 |
| **Glycogen storage diseases** | | | | | | | | | |
| [Kishnani PS-2014](http://www.ncbi.nlm.nih.gov/pubmed/?term=Kishnani PS%5BAuthor%5D&cauthor=true&cauthor_uid=25356975) | SR | 86 | 61 | 47 | 100 | 58 | 63 | 75 | 2.5 |
| Kishnani PS-2010 | R | 89 | 61 | 50 | 83 | 48 | 33 | 67 | 2 |
| [Rake JP](http://www.ncbi.nlm.nih.gov/pubmed/?term=Rake JP%5BAuthor%5D&cauthor=true&cauthor_uid=12373584) | R | 94 | 17 | 27 | 92 | 25 | 0 | 58 | 2 |
| [Visser G](http://www.ncbi.nlm.nih.gov/pubmed/?term=Visser G%5BAuthor%5D&cauthor=true&cauthor_uid=12373585) | R | 83 | 19 | 23 | 83 | 23 | 0 | 50 | 2 |
| **Hemochromatoses** | | | | | | | | | |
| Bacon BR | R | 56 | 44 | 75 | 83 | 46 | 17 | 67 | 2 |
| BCMA | R | 94 | 8 | 13 | 81 | 27 | 0 | 58 | 2 |
| Qaseem A | NR | 100 | 36 | 27 | 50 | 23 | 42 | 33 | 1.5 |
| **Gaucher disease** | | | | | | | | | |
| Andersson | R | 83 | 33 | 29 | 67 | 25 | 0 | 58 | 1.5 |
| [Charrow J](http://www.ncbi.nlm.nih.gov/pubmed/?term=Charrow J%5BAuthor%5D&cauthor=true&cauthor_uid=14722528) | R | 81 | 56 | 43 | 69 | 33 | 13 | 58 | 2 |
| [Grabowski GA](http://www.ncbi.nlm.nih.gov/pubmed/?term=Grabowski GA%5BAuthor%5D&cauthor=true&cauthor_uid=14677061) | R | 72 | 33 | 27 | 56 | 33 | 0 | 50 | 2 |
| [Kaplan P](http://www.ncbi.nlm.nih.gov/pubmed/?term=Kaplan P%5BAuthor%5D&cauthor=true&cauthor_uid=22772880) | R | 78 | 39 | 29 | 78 | 38 | 100 | 83 | 2 |
| Vellodi A | NR | 83 | 61 | 30 | 72 | 17 | 0 | 42 | 2.5 |
| [Vom Dahl S](http://www.ncbi.nlm.nih.gov/pubmed/?term=Vom Dahl S%5BAuthor%5D&cauthor=true&cauthor_uid=16846538) | R | 89 | 36 | 54 | 56 | 31 | 83 | 50 | 2 |
| Weinreb NJ | R | 67 | 39 | 30 | 67 | 17 | 4 | 50 | 2 |
| **Fabry disease** | | | | | | | | | |
| Bennett RL | SR | 92 | 86 | 69 | 78 | 52 | 46 | 83 | 3 |
| Biegstraaten M | SR | 64 | 81 | 65 | 64 | 42 | 71 | 67 | 2.5 |
| [Desnick RJ](http://www.ncbi.nlm.nih.gov/pubmed/?term=Desnick RJ%5BAuthor%5D&cauthor=true&cauthor_uid=12585833) | NR | 64 | 22 | 40 | 53 | 48 | 46 | 42 | 2 |
| Eng CM | R | 92 | 42 | 45 | 89 | 46 | 0 | 67 | 2 |
| Kes VB | R | 61 | 33 | 29 | 56 | 21 | 0 | 50 | 2 |
| Laney DA | R | 94 | 92 | 46 | 78 | 46 | 83 | 67 | 2.5 |
| Ortiz A | R | 58 | 31 | 40 | 61 | 23 | 92 | 50 | 2.5 |
| [Terryn W](http://www.ncbi.nlm.nih.gov/pubmed/?term=Terryn W%5BAuthor%5D&cauthor=true&cauthor_uid=23234755) | SR | 81 | 44 | 67 | 78 | 58 | 54 | 67 | 2.5 |
| **Mucopolisaccharidoses** | | | | | | | | | |
| [De Ru MH](http://www.ncbi.nlm.nih.gov/pubmed/?term=de Ru MH%5BAuthor%5D&cauthor=true&cauthor_uid=21831279) | SR | 89 | 50 | 63 | 89 | 54 | 58 | 75 | 2.5 |
| Fahnehjelm KT | R | 75 | 58 | 32 | 81 | 38 | 50 | 75 | 2.5 |
| Giugliani R | R | 75 | 56 | 56 | 67 | 29 | 38 | 58 | 2 |
| Langereis EJ | R | 86 | 72 | 58 | 53 | 23 | 46 | 58 | 2 |
| [Muenzer J -2012](http://www.ncbi.nlm.nih.gov/pubmed/?term=Muenzer J%5BAuthor%5D&cauthor=true&cauthor_uid=22037758) | R | 92 | 53 | 33 | 69 | 40 | 92 | 50 | 2.5 |
| Muenzer J-2009 | R | 69 | 36 | 28 | 69 | 42 | 50 | 58 | 2 |
| Scarpa M | SR | 61 | 75 | 61 | 89 | 42 | 58 | 75 | 2.5 |
| Solanki GA | R | 72 | 39 | 24 | 61 | 54 | 100 | 50 | 1.5 |
| OR: overall recommendation; D: domain; OQA: overall quality assessment; GR: grade of recommendation; BCMA: British Columbia Medical Association. SR: strongly recommended; R: recommended; NR: not recommended. Domain scores were calculated as described in Methods. | | | | | | | | | |
